# Activated FGFR3 suppresses bone regeneration and bone mineralization in an ovariectomized mouse model

**DOI:** 10.1186/s12891-023-06318-9

**Published:** 2023-03-16

**Authors:** Itaru Kawashima, Masaki Matsushita, Kenichi Mishima, Yasunari Kamiya, Yusuke Osawa, Bisei Ohkawara, Kinji Ohno, Hiroshi Kitoh, Shiro Imagama

**Affiliations:** 1 Department of Orthopaedic Surgery, Nagoya University Graduate School of Medicine, 4668550 Nagoya, Aichi Japan; 2 Division of Neurogenetics, Center for Neurological Diseases and Cancer, Nagoya University Graduate School of Medicine, 4668550 Nagoya, Aichi Japan; 3 Department of Orthopaedic Surgery, Aichi Children’s Health and Medical Center, 4748710 Obu, Aichi Japan; 4 Department of Comprehensive Pediatric Medicine, Nagoya University Graduate School of Medicine, 4668550 Nagoya, Aichi Japan

**Keywords:** Postmenopausal osteoporosis, Fibroblast growth factor receptors 3, FGFR3, Distraction osteogenesis, Ovariectomy, Meclozine

## Abstract

**Background:**

Postmenopausal osteoporosis is a widespread health concern due to its prevalence among older adults and an associated high risk of fracture. The downregulation of bone regeneration delays fracture healing. Activated fibroblast growth factor receptor 3 (FGFR3) accelerates bone regeneration at juvenile age and downregulates bone mineralization at all ages. However, the impact of FGFR3 signaling on bone regeneration and bone mineralization post-menopause is still unknown. This study aimed to evaluate the impact of FGFR3 signaling on bone regeneration and bone mineralization during menopause by developing a distraction osteogenesis (DO) mouse model after ovariectomy (OVX) using transgenic mice with activated FGFR3 driven by *Col2a1* promoter (*Fgfr3* mice).

**Methods:**

The OVX or sham operations were performed in 8-week-old female *Fgfr3* and wild-type mice. After 8 weeks of OVX surgery, DO surgery in the lower limb was performed. The 5-day-latency period followed by performing distraction for 9 days. Bone mineral density (BMD) and bone regeneration was assessed by micro-computed tomography (micro-CT) scan and soft X-ray. Bone volume in the distraction area was also evaluated by histological analysis after 7 days at the end of distraction. Osteogenic differentiation and mineralization of bone marrow-derived mesenchymal stem cells (BMSCs) derived from each mouse after 8 weeks of the OVX or sham operations were also evaluated with and without an inhibitor for FGFR3 signaling (meclozine).

**Results:**

BMD decreased after OVX in both groups, and it further deteriorated in *Fgfr3* mice. Poor callus formation after DO was also observed in both groups with OVX, and the amount of regenerated bone was further decreased in *Fgfr3* mice. Similarly, histological analysis revealed that *Fgfr3* OVX mice showed lower bone volume. Osteogenic differentiation and mineralization of BMSCs were also deteriorated in *Fgfr3* OVX mice. An inhibitor for FGFR3 signaling dramatically reversed the inhibitory effect of OVX and FGFR3 signaling on BMSC mineralization.

**Conclusion:**

Upregulated FGFR3 decreased newly regenerated bone after DO and BMD in OVX mice. FGFR3 signaling can be a potential therapeutic target in patients with postmenopausal osteoporosis.

**Supplementary Information:**

The online version contains supplementary material available at 10.1186/s12891-023-06318-9.

## Background

Osteoporosis is a common skeletal disease among older adults and involves a high risk of fracture owing to bone fragility [1]. Since bone loss further deteriorates after menopause [2], osteoporosis is more common in women than in men. The prevalence of osteoporosis increases with age, and 20% women aged ≥ 40 years have been diagnosed with osteoporosis [3]. Therefore, postmenopausal osteoporosis is a widespread issue. In addition to increasing the risk of fracture, postmenopausal osteoporosis causes delayed fracture healing [4], which occasionally compromises the patients’ quality of life due to chronic pain. Female animals subjected to ovariectomy (OVX) (whose ovaries are resected) have been developed to investigate the osteoporotic condition [5]. Rats and mice developed after OVX have been employed to replicate human postmenopausal osteoporosis [6, 7]. Impaired fracture healing has been demonstrated in an OVX rat model using radiological and histological analyses [6, 8]. Similarly, newly regenerated bone in rats after OVX is significantly decreased after distraction osteogenesis (DO) [9], which is a unique technique for promoting bone regeneration.

Since the fibroblast growth factor receptor 3 (FGFR3) gene is a negative regulator of endochondral bone development [10, 11], and gain-of-function mutations of FGFR3 lead to a short-limbed short stature. In some countries, patients with short stature often have a limb lengthening surgery by DO. Superior bone regeneration during DO has been indicated in FGFR3-related skeletal dysplasia compared to that in other etiologies, among adolescent and young adult patients [12]. We have previously demonstrated accelerated bone regeneration after DO, with increased numbers of both osteoblasts and osteoclasts, in a mouse model with activated FGFR3 driven by *Col2a1* promoter at the age of 4 weeks [13]. In contrast, trabecular bone architecture and bone mineral density (BMD) had been deteriorated via downregulating bone mineralization in a transgenic mouse with a gain-of-function mutation in FGFR3 from 2 to 4 months of age [14]. Thus, activated FGFR3 signaling can upregulate new bone formation in juvenile age, but downregulate bone mineralization throughout all ages. However, the impact of FGFR3 signaling on bone regeneration and bone mineralization post-menopause is still unknown. This study aimed to assess the influence of FGFR3 signaling on the ability of bone regeneration and bone mineralization during menopause using a mouse model of DO mimicking postmenopausal osteoporosis.

## Methods

### Mice

*Fgfr3*^ach/+^ mice (FVB background) were kindly provided by Dr. David M. Ornitz of the Washington University [15]. These conditional transgenic mice (hereafter designated as *Fgfr3* mice) had a heterozygous p.G380R mutation in the *Fgfr3* gene, leading to gain-of-function of FGFR3 signaling, which was achieved under the control of the *Col2a1* promoter. The mice were housed under a 12 h light–dark cycle and given free access to feed (a standard commercial diet) and water. All experiments were carried out in accordance with protocols approved by the Animal Care and Use Committee of Nagoya University Graduate School of Medicine.

### Surgery and distraction protocol

**Fig. 1:**
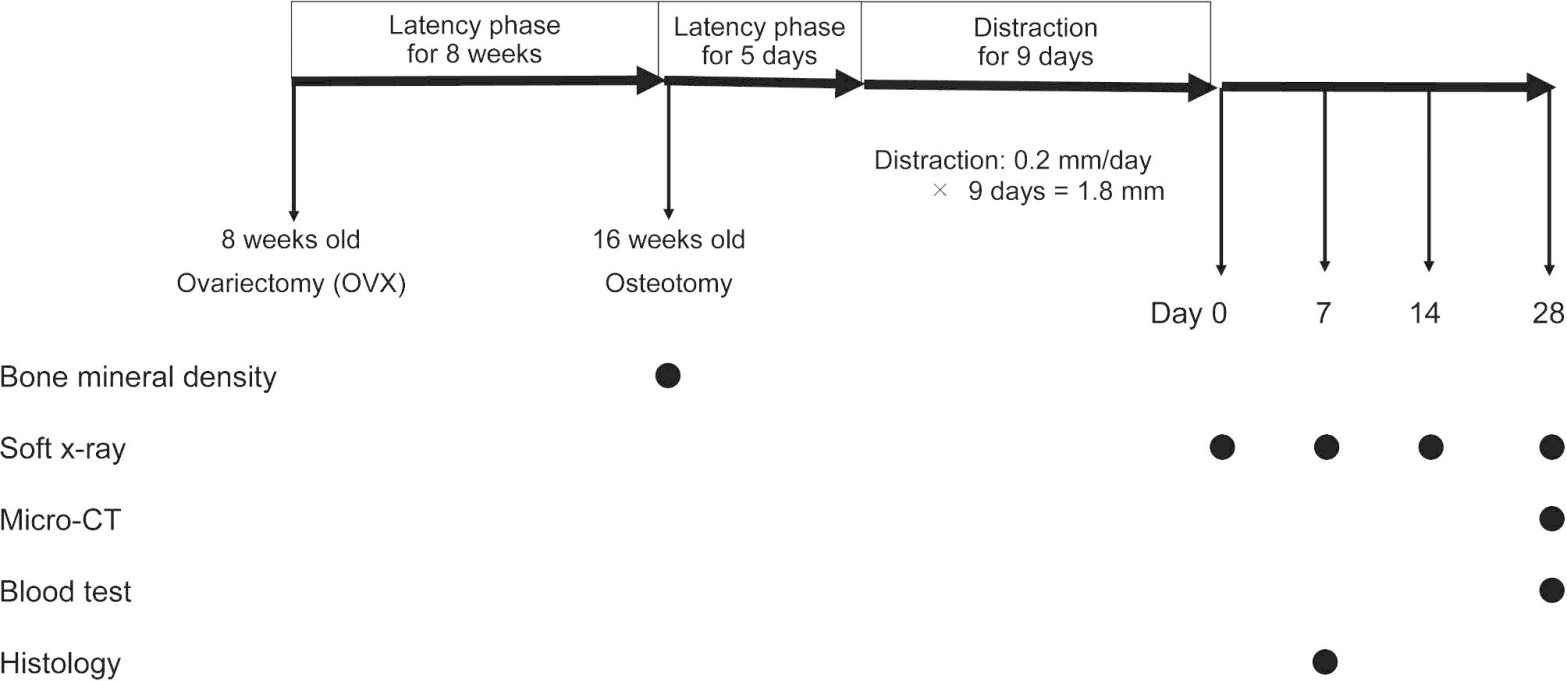
Experimental Design. Ovariectomy (OVX) and tibial osteotomy (distraction osteogenesis [DO] surgery) were performed at the ages of 8 and 16 weeks, respectively. After osteotomy, a 5-day-latency phase was followed by distraction at a rate of 0.2 mm/day for 9 days. Day 0 was defined as the day of distraction completion. Closed circles indicate the time points for each analysis including bone mineral density, soft X-ray, micro-computed tomography (CT), blood test, and histology

OVX was performed in 8-week-old female *Fgfr3* mice and wild-type mice (FVB background), according to established protocols (Fig. 1) [5, 7]. Briefly, under isoflurane anesthesia, the ovary and the oviduct were rapidly removed by the bilateral dorsal abdominal approach. Each ovary was excised at the tip of the uterine horn. In the sham procedure, a similar incision was made, and the ovary was visualized, but no tissue was removed. Mice were randomly subjected to OVX or sham surgery, and assigned to four groups, namely *Fgfr3* OVX, *Fgfr3* sham, wild-type OVX, and wild-type sham.

After 8 weeks of OVX or sham surgery, the mouse model of DO in the lower limb was produced in all groups using a previously described method [16]. Briefly, an anterior longitudinal incision was made on the left lower leg under isoflurane anesthesia. After fibulectomy, 27-gauge needles were inserted at both ends of the tibia. These needles were then fixed with the external fixator consisting of two incomplete acrylic resin rings and an expansion screw (Ortho Dentaurum). After complete polymerization, osteotomy was performed at the middle of the diaphysis in the tibia. The wound was closed with a 5–0 nylon suture. The protocol consisted of 5 days of latency period followed by distraction at a rate of 0.2 mm every 24 h for 9 days. Day 0 was defined as the day at the end of distraction. We performed DO on 27 *Fgfr3* mice and 45 wild-type mice (Additional Fig. 1 ). Among these, 16 *Fgfr3* mice (n = 8 each in OVX and sham group) and 23 wild-type mice (OVX group, n = 11; sham group, n = 12) were subjected to radiographic evaluation using soft X-ray at days 0, 7, 14, and 28 under general anesthesia and sacrificed at day 28 to perform micro-computed tomography (micro-CT) scan and blood test. The remaining 11 *Fgfr3* mice (OVX group, n = 6; sham group, n = 5) and 22 wild-type mice (OVX group, n = 11; sham group, n = 11) were subjected to BMD analysis after 8 weeks of OVX and sacrificed at day 7 for histological evaluation. Additionally, 20 *Fgfr3* mice and 24 wild-type mice were subjected to cell culture. Thus, we employed a total of 47 *Fgfr3* mice and 69 wild-type mice in the current study.

### Morphological analysis

Under isoflurane anesthesia, the mice were subjected to a soft X-ray (30 kV, 5 mA for 20 s; SOFTEX Type CMB-2; SOFTEX) at 0, 7, 14, and 28 days after completion of distraction. The callus formations were quantified using bone fill scores (i.e., 0, 1, 2, and 3 represent 0, 0–50, 50–100, and 100% bone fills, respectively) [17, 18]. Based on lateral radiographic images taken on day 28, the number of uniting callus was assessed by the formation of a seamless bridging callus according to the previous study [19]. The number of uniting callus was counted: score 0 represents no union of both anteroposterior cortices, score 1 represents union of either anterior or posterior hemicortex, and score 2 represents union of both anteroposterior cortices.

Micro-CT (Al ± Cu filter, voxel size 0.9 μm, 80 kV, 313 µA for 0.203 s; SkyScan1176, Bruker) examinations were performed on day 28. After reconstruction using the SkyScan NRecon software, the images were analyzed using three-dimensional (3D) algorithms in SkyScan CTAn software, according to the manufacturer’s instructions. The region of interest was determined as the distraction region surrounded by the outlined periosteum from the proximal and distal ends, according to the previous studies [20, 21], and bone volume (BV) and BV to tissue volume ratio (BV/TV) were measured. BMD of the proximal tibia was also quantified 8 weeks after OVX or sham surgery. Calibration was performed using a phantom before the measurement of the tibia. The position for micro-CT scanning was 1 to 3 mm below the growth plate from the proximal tibia.

### Histological analysis

At day 7, the distracted tibiae were stained using Villanueva Goldner staining. Specimens were fixed with 4% paraformaldehyde and embedded in methyl methacrylate without decalcification (Kureha Special Laboratory). Calcified and osteoid areas were quantified using the Image J software, according to the previous studies [22, 23], in a blinded manner. BV/distraction area and osteoid volume (OV)/distraction area were determined, according to the previous studies [19, 22, 24]. For immunohistochemistry, the sections were stained with antibodies specific for tartrate-resistant acid phosphatase (TRAP) after embedding in glycolmethacrylate without decalcification (Kureha Special Laboratory). The most central sections of the medullary cavity were chosen for histomorphometric analyses. Three arbitrary parts of newly regenerated bone were chosen for counting the number of osteoclasts in TRAP staining.

### Serum biochemistry and immunoassay

Serum was obtained from the mice on day 28 to analyze total calcium, phosphate, and alkaline phosphatase (ALP) using an automatic analyzer (Fuji Dri-Chem, Fujifilm), and receptor activator of nuclear factor kappa-Β ligand (RANKL) levels using enzyme-linked immunosorbent assay (ELISA) (R&D Systems).

### Cell culture

Bone marrow-derived mesenchymal stem cells (BMSCs) were derived from *Fgfr3* mice and wild-type mice at the age of 4 weeks (*Fgfr3* group, n = 4; wild-type group, n = 4) and at 8 weeks after the OVX (*Fgfr3* group, n = 8; wild-type group, n = 10) or sham (*Fgfr3* group, n = 8; wild-type group, n = 10) surgery (i.e., at the age of 16 weeks). The bone marrows were flushed from both sides of the tibiae and femurs under aseptic conditions using Dulbecco’s modified Eagle’s medium (Merck) containing 10% fetal bovine serum (Gibco) and 1% penicillin/streptomycin (Thermo Fischer). Cells were cultured in a humidified atmosphere of 5% CO_2_ at 37 ℃. All assays were carried out on second passage cultures. For the proliferation assay, cells were seeded at 2 × 10^6^ cells in a 100 mm dish and cultured for 5 days. To evaluate the osteogenic differentiation, cells were seeded at 1 × 10^5^ cells in a 24-multiwell plate. On the next day of seeding, the medium was replaced with fresh medium supplemented with 50 µg/mL of ascorbic acid, 10 mM beta-glycerophosphate, and 10^− 7^ M dexamethasone (Merck) with vehicle or with 20 µM meclozine, which is an inhibitor of FGFR3 signaling [25, 26]. The medium was replaced on the next day of the seeding and repeated every three days. ALP activity staining (Cosmo Bio LTD) and Alizarin red staining (Merck) were performed on days 7 or 21, and the stained areas were measured using ImageJ Fiji software.

### Statistical analyses

All statistical analyses were performed using IBM SPSS Statistics version 27 (IBM). Statistical analyses were carried out using one-way analysis of variance (ANOVA), or two-way repeated-measures ANOVA with post-hoc Bonferroni correction for comparison among four groups and Student’s t-test for comparison between two groups, and statistical significance was set at p < 0.05.

## Results

### OVX further deteriorated the low BMD in Fgfr3 mice

**Fig. 2:**
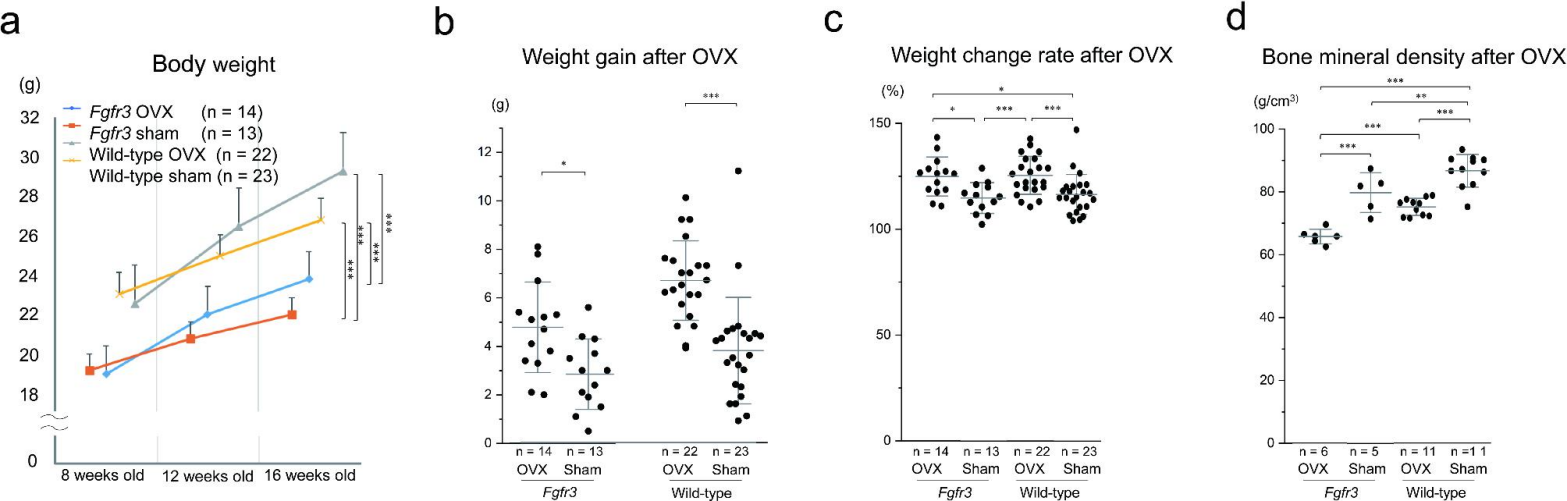
Body weight and bone mineral density (BMD) after ovariectomy (OVX) in each group. **a** Body weight before and after OVX, which was performed at the age of 8 weeks. Data values are presented as means and standard error (SE). **b** Weight gain 8 weeks after OVX compared to that at the age of 8 weeks. Dots indicate the value of each sample and bars indicate the means and standard deviation (SD). **c** Weight change rate indicating the relative weight 8 weeks after OVX. Dots indicate the value of each sample and bars indicate the means and SD. **d** BMD after 8 weeks of OVX. Dots indicate the value of each sample and bars indicate the means and SD. Statistical significance was analyzed using two-way **(a)** or one-way **(b and c)** analysis of variance (ANOVA) with post-hoc Bonferroni correction. Statistical significance was expressed as * p < 0.05, * p < 0.01 and *** p < 0.005

At the age of 8 weeks, body weight was higher in wild-type mice than that in *Fgfr3* mice (p < 0.005). OVX significantly increased average body weights in, both, wild-type and *Fgfr3* mice after 4 and 8 weeks of the surgery (Fig. 2a-c). At the age of 16 weeks, BMD was significantly decreased in *Fgfr3* mice compared to that in wild-type mice (p < 0.01), and OVX further deteriorated the BMD in *Fgfr3* mice (p < 0.005) (Fig. 2d).

### **Callus formation** deteriorated **in*Fgfr3*OVX mice**

**Fig. 3:**
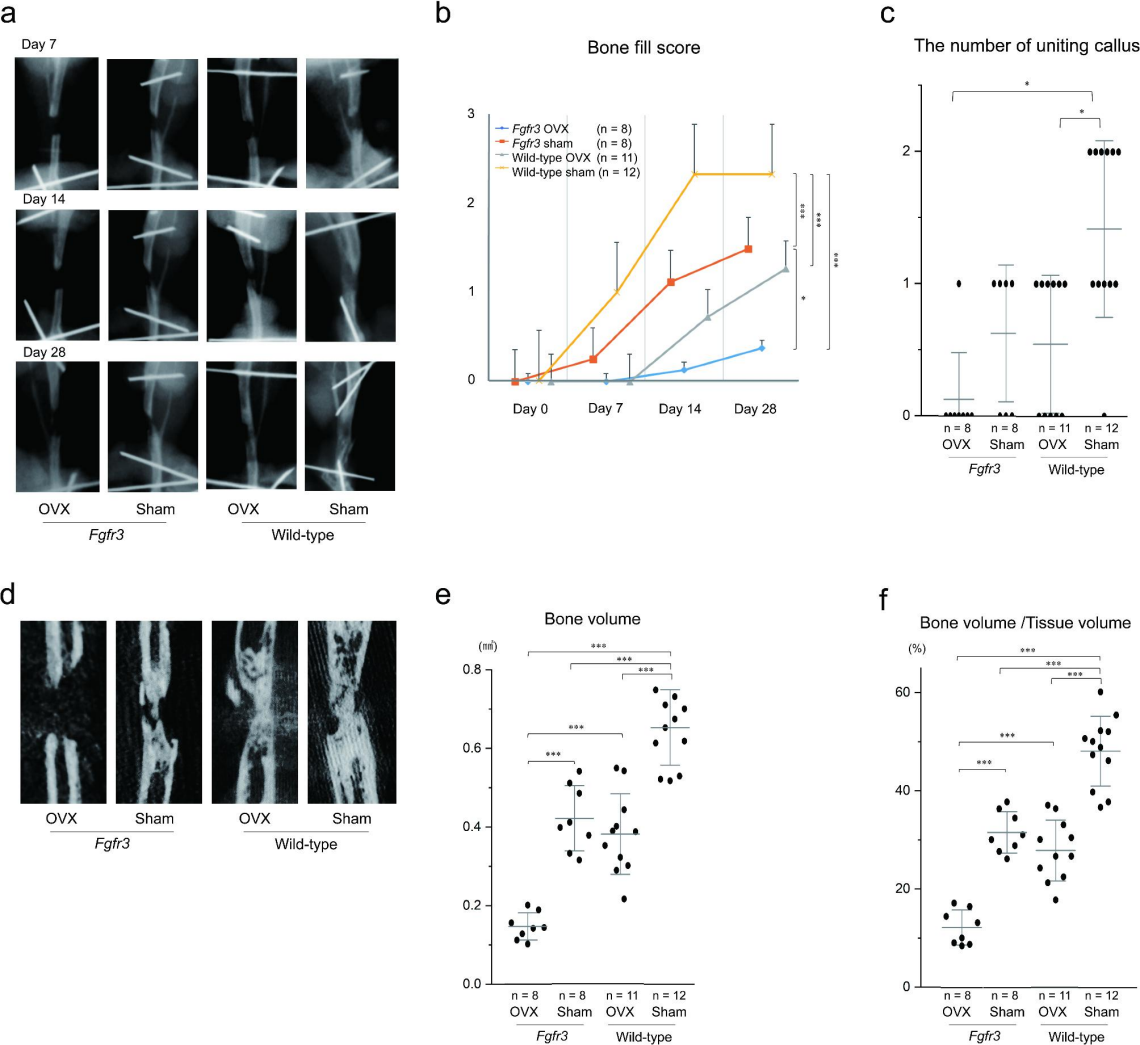
Callus formation after distraction osteogenesis (DO). **a** Representative radiograph in each group at days 7, 14, and 28. **b** Bone fill score to quantify bony calluses at days 0, 7, 14, and 28. Data values are presented as means and standard error (SE). **c** Number of uniting calluses on day 28. Dots indicate the value of each sample and bars indicate the means and standard deviation (SD). **d** Representative micro-computed tomographic scan images in each group on day 28. **e** Bone volume (BV) of the distraction area in each group. Dots indicate the value of each sample and bars indicate the means and SD. **f** Bone volume (BV)/Tissue volume (TV) of the distraction area in each group. Dots indicate the value of each sample and bars indicate the means and SD. Statistical significance was analyzed using two-way **(b)** or one-way analysis of variance (ANOVA) **(c, e, and f)** with post-hoc Bonferroni correction. Statistical significance was expressed as * p < 0.05 and *** p < 0.005

We performed radiological analysis at each time point after DO using soft X-rays. Callus formation gradually increased in each mouse during the consolidation phase, except for that in *Fgfr3* OVX mice (Fig. 3a). The bone fill score of *Fgfr3* mice was significantly lower than that of wild-type mice at days 7, 14, and 28, respectively (Fig. 3b). OVX further decreased bone fill scores in mice of both groups. We additionally measured the number of united calluses at day 28 and found that *Fgfr3* OVX mice had less united calluses compared to those in other groups (Fig. 3c).

Further, we evaluated callus formation using micro-CT scanning. On day 28, callus formation apparently decreased in *Fgfr3* OVX mice (Fig. 3d). Both BV and BV/TV of the distraction area were significantly lower in *Fgfr3* OVX mice than those in mice without OVX (Fig. 3e and f).

### **Osteoblasts were** downregulated **in the newly formed bone of*Fgfr3*mice after OVX**

**Fig. 4:**
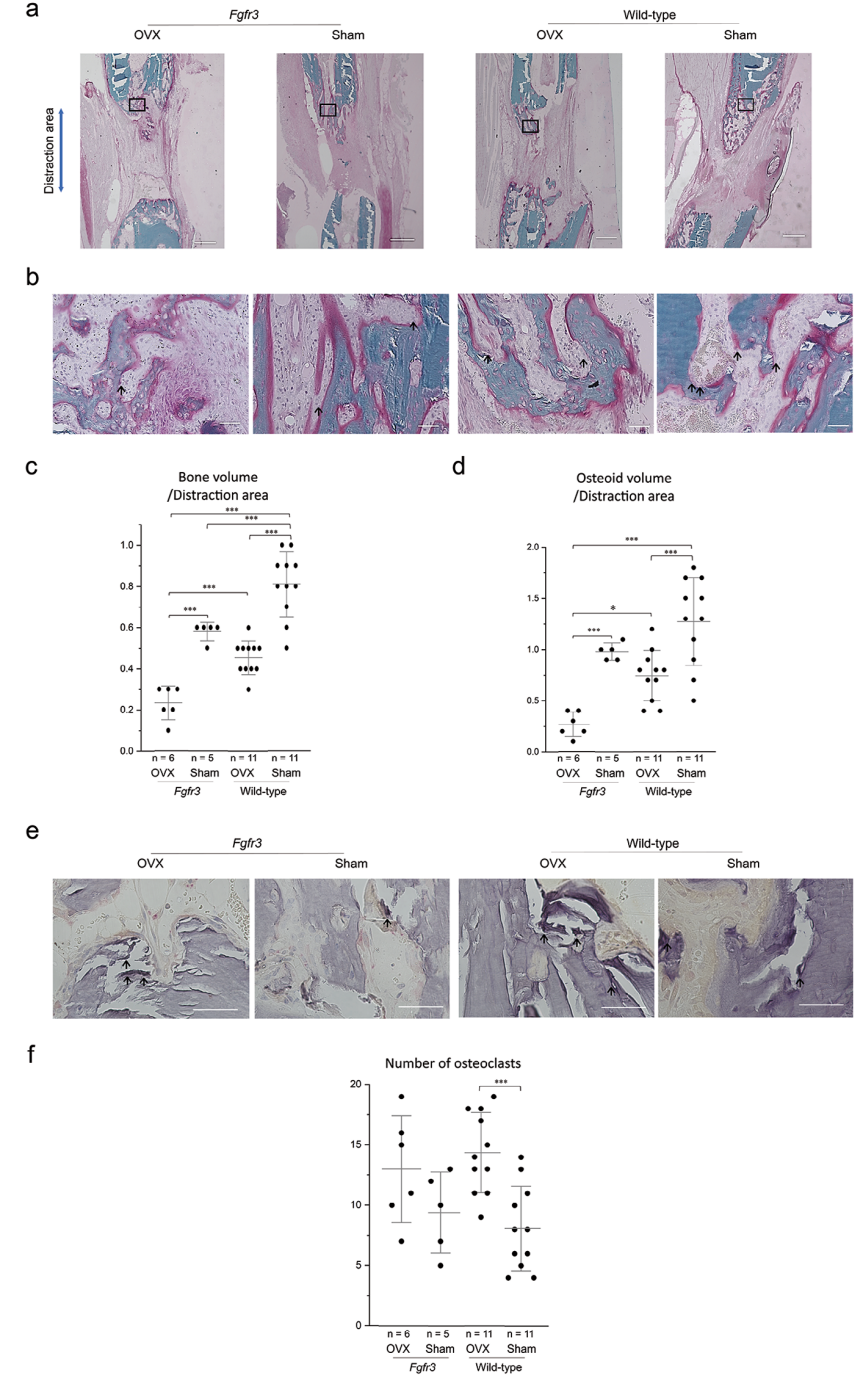
Histological evaluation of the distraction area 7 days after distraction osteogenesis (DO). **a** Representative undecalcified histology of distracted area stained with Villanueva Goldner staining. Green signals show calcified bones. Red signals show osteoid bones. Squared parts are magnified in panel **b**. Scale bare indicates 500 μm. **b** High magnification images in the central part of the newly formed bone in each group. Arrow shows osteoblasts around osteoid bone. Scale bare indicates 50 μm. **c** Dots indicate the value of each sample and bars indicate the mean and standard deviation (SD) of Bone volume (BV)/distraction area. **d** Dots indicate the value of each sample and bars indicate the mean and SD of osteoid volume/distraction area. **e** Representative high magnification images of tartrate-resistant acid phosphatase (TRAP) staining in the central part of the newly formed bone. Purple signals show osteoclasts. **f** Dots indicate the value of each sample and bars indicate the mean and SD of the number of osteoclasts. Statistical significance was analyzed using one-way analysis of variance (ANOVA) with post-hoc Bonferroni correction. Statistical significance was expressed as * p < 0.05 and *** p < 0.005

Further, we histologically evaluated the distraction area stained with Villanueva Goldner on day 7. Similar to the radiological findings, less callus formation was observed in *Fgfr3* OVX mice (Fig. 4a). The BV and OV per distraction area were significantly decreased in *Fgfr3* mice compared to those in wild-type mice, and OVX further deteriorated these parameters (Fig. 4c and d). High magnification images of the newly regenerated bone’s central region revealed smaller number of osteoblasts in *Fgfr3* OVX mice, while more osteoblasts were observed surrounding the osteoid in wild-type sham mice (Fig. 4b). There were increased number of osteoclasts in the newly regenerated bone after OVX in the wild-type mice (Fig. 4e and f).

### **ALP and RANKL were** upregulated **after OVX in*Fgfr3*and wild-type mice**

**Fig. 5:**
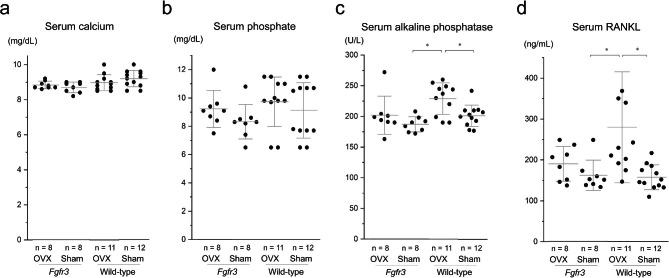
Serum biochemistry and immunoassay in each group 28 days after distraction osteogenesis (DO). Serum levels of **a** total calcium, **b** phosphate, **c** alkaline phosphatase (ALP), and **d** receptor activator of nuclear factor kappa-Β ligand (RANKL). Dots indicate the value of each sample and bars indicate the means and standard deviation (SD). Statistical significance was analyzed using one-way analysis of variance (ANOVA) with post-hoc Bonferroni correction. Statistical significance was expressed as * p < 0.05

At day 28, there were no significant differences in serum calcium and phosphate levels between mice in the *Fgfr3* and wild-type groups (Fig. 5a and b). In wild-type mice, serum ALP and RANKL levels were higher after OVX than those in the sham group; however, there were no statistical differences in *Fgfr3* OVX and sham mice (Fig. 5c and d).

### Osteogenic differentiation and mineralization of BMSCs deteriorated in *Fgfr3* OVX mice

**Fig. 6:**
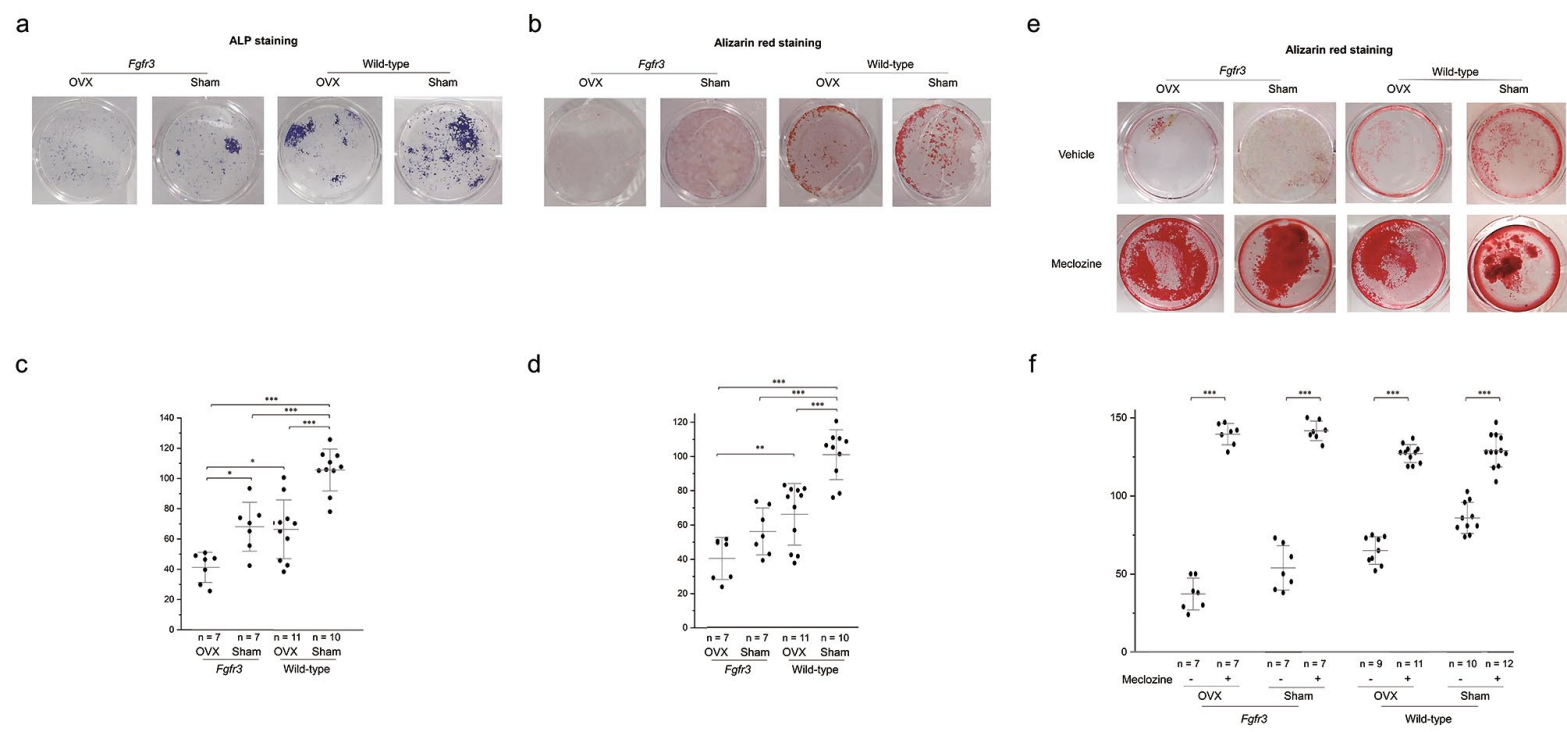
Osteogenic differentiation and mineralization of bone marrow-derived mesenchymal stem cells (BMSCs) in each group. **a** Representative result of alkaline phosphatase (ALP) staining for cells in cultured BMSCs in each group. **b** Quantitative analysis of ALP activity. Dots indicate the value of each sample and bars indicate the means and standard deviation (SD). **c** Representative result of Alizarin red staining of the mineralized osteoblasts in each group. **d** Quantitative analysis of Alizarin red staining. Dots indicate the value of each sample and bars indicate the means and SD. **e** Representative result of Alizarin red staining for cultured BMSCs with meclozine treatment. **f** Quantitative analysis of Alizarin red staining to assess the mineralization of BMSCs. Dots indicate the value of each sample and bars indicate the means and SD. Statistical significance was analyzed using one-way analysis of variance (ANOVA) with post-hoc Bonferroni correction. Statistical significance was expressed as * p < 0.05, ** p < 0.01 and *** p < 0.005

To evaluate the effect of OVX and FGFR3 on the osteogenic differentiation and mineralization of BMSCs, we compared the results of ALP and Alizarin red stainings of BMSCs among *Fgfr3* and wild-type mice with or without OVX. After osteogenic culture for 21 days, both, ALP and Alizarin red stainings were apparently reduced in *Fgfr3* mice compared to those in wild-type mice, and further deteriorated after OVX (Fig. 6a and c). Quantitative analyses revealed that both the stains were significantly reduced in *Fgfr3* OVX mice (Fig. 6b and d). Considering 4-week-old mice, we further performed ALP and Alizarin red stainings of BMSCs. On the seventh day of the culture, ALP staining was upregulated in 4-week-old *Fgfr3* mice, contrary to the results obtained in 16-week-old mice (Additional Fig. 1a and b). In contrast, Alizarin red staining after the 21-day culture was less in *Fgfr3* mice than in wild-type mice at the age of 4 weeks (Additional Fig. 1c and d). Next, we evaluated the effect of meclozine, an inhibitor of FGFR3 signaling, employing the drug repositioning strategy [25, 26] on the mineralization of BMSCs in the *Fgfr3* OVX mice. We administered meclozine to the BMSCs of the *Fgfr3* and wild-type mice with or without OVX. After meclozine treatment, Alizarin red staining was surprisingly increased and there was apparently no difference in the staining between *Fgfr3* and wild-type mice with or without OVX (Fig. 6e). Quantitative analysis revealed that meclozine significantly increased Alizarin red staining in all the groups (Fig. 6f). On the other hand, ALP staining was decreased after meclozine treatment in all groups except for *Fgfr3* OVX mice (Additional Fig. 1a and b).

## Discussion

The present study showed that the ability of bone regeneration after DO and BMD were decreased in OVX mice, similar to the findings of a previous study using a rat model of DO [9]. We found that activated FGFR3 suppressed bone regeneration and deteriorated the thin trabecular architecture in 16-week-old OVX mice. Histological analysis of the newly regenerated bone revealed that osteoblasts were downregulated and osteoclasts were upregulated after OVX. The osteoblasts were further decreased in *Fgfr3* mice. As a previous study demonstrated [27], OVX suppressed the osteogenic differentiation and mineralization of BMSCs. These were further deteriorated in the combination of *Fgfr3* and OVX mice in the current study. Meclozine, an inhibitor of FGFR3 signaling [25, 26], reversed the mineralization of BMSCs in *Fgfr3* OVX mice, although the mineralization was poor without meclozine treatment in both *Fgfr3* and wild-type mice.

A mouse model of gain-of-function mutation in FGFR3 has shown reduced bone mineralization [14]. FGFR3 activation in immature osteoblasts has led to lower mineralization activity owing to defective bone remodeling in 3-month-old mice [28]. Col2a1 is transiently expressed in immature osteoblasts during early development as well as adulthood [29, 30]. Since FGFR3 was overexpressed under the Col2 promoter in *Fgfr3* mice in this study, bone mineralization could be downregulated via FGFR3-activated immature osteoblasts. In contrast, *Fgfr3* deletion in osteoclast lineage cells of mice inhibits bone resorption [31]. This indicates that activated FGFR3 promotes bone resorption by enhancing osteoclastogenesis. However, differences in osteoclastogenesis were not observed between *Fgfr3* and wild-type mice, since the effect of *Fgfr3* on promoting osteoclastogenesis might not be manifested probably due to the effect of DO surgery (i.e., DO surgery could recruit the osteoclasts after increasing osteoblasts [32]).

In 4-week-old *Fgfr3* mice, bone regeneration after DO was accelerated with increased numbers of osteoblasts [13]. Our previous results were contrary to the current results using 16-week-old *Fgfr3* mice. These differences could be attributed to the ALP activity, which was upregulated at 4 weeks but downregulated at 16 weeks in *Fgfr3* mice. Although bone regeneration in young patients with FGFR3-related skeletal dysplasias, including achondroplasia and hypochondroplasia, is usually enhanced, aging and post-menopause would lead to poor bone healing in addition to low BMD in these patients.

Interestingly, meclozine, which inhibits Erk1/2 phosphorylation in FGFR3 signaling [25], dramatically improved the mineralization of BMSCs, particularly in *Fgfr3* OVX mice. Similar to the results of an Erk1/2 inhibitor (PD98059) [14], meclozine could reverse the inhibitory effect of FGFR3 on bone mineralization. Since meclozine also improves bone loss in the OVX mice via inhibiting Erk1/2 phosphorylation of RANKL signaling in osteoclasts [33], the synergistic effect of meclozine on inhibiting both FGFR3 and RANKL signaling would reverse bone mineralization in the current BMSCs. To assess the effect of FGFR3 inhibitors on bone regeneration and bone mineralization during menopause, an *in-vivo* study employing *Fgfr3* OVX mice is required. The optimal timing and duration of administrating FGFR3 inhibitors may be considered during DO to develop treatment strategies of fracture healing associated with postmenopausal osteoporosis.

This study had certain limitations that warrant discussion. First, we did not conduct the analysis of osteoclast function using a cell model of activated FGFR3 signaling, although the inhibitory effect of meclozine on RANKL signaling has been demonstrated employing primary bone marrow-derived macrophages [33]. Therefore, we speculate that meclozine may inhibit RANKL signaling in addition to FGFR3 signaling. An in vitro study of osteoclast function may accurately assess the impact of meclozine on activated FGFR3 signaling in postmenopausal osteoporosis. Second, DO surgery influenced the results of biochemical parameters, including serum calcium, phosphate, ALP, and RANKL. Mice that do not undergo DO surgery may show different results. We evaluated the osteogenic differentiation and mineralization of BMSCs without DO surgery using ALP and Alizarin red stainings and the results obtained were similar to the morphological and histological analysis for bone regeneration. Third, the mechanism of fluctuation in ALP activation with age remained unclear in *Fgfr3* mice. Lastly, we did not conduct the animal study of DO using inhibitors of FGFR3 signaling, although there have been several animal studies in which meclozine ameliorated bone mass in OVX mice [33] as well as a juvenile mouse model of FGFR3-related skeletal dysplasia [26]. To assess the effect of FGFR3 inhibitors on bone regeneration and bone mineralization during DO, considering the optimal dose and timing of administrating these inhibitors is required. Thus, the colossally conceived experiment will be conducted as another study in the future.

## Conclusion

Activated FGFR3 suppresses the ability of bone regeneration and bone mineralization in a mouse model of postmenopausal osteoporosis. Thus, FGFR3 signaling could be a potential therapeutic target for patients with postmenopausal osteoporosis.

## Electronic supplementary material

Below is the link to the electronic supplementary material.

Supplementary Material 1

## Data Availability

The datasets of the current study are available from the corresponding authors on request.

## List of abbreviations

FGFR3: fibroblast growth factor receptor 3
DO: distraction osteogenesis
OVX: ovariectomy
BMSC: bone marrow-derived mesenchymal stem cell
BMD: bone mineral density
CT: computed tomography
3D: three-dimensional
BV: bone volume
TV: tissue volume
OV: osteoid volume
TRACP: tartrate-resistant acid phosphatase
ALP: alkaline phosphatase
RANKL: receptor activator of nuclear factor kappa-Β ligand
ELISA: enzyme-linked immunosorbent assay
ANOVA: analysis of variance

## References

[1] Kanis JA, Johnell O, Oden A, Johansson H, McCloskey E (2008). FRAX and the assessment of fracture probability in men and women from the UK. Osteoporos Int.

[2] Li L, Wang Z (2018). Ovarian aging and osteoporosis. Adv Exp Med Biol.

[3] Wang L, Yu W, Yin X, Cui L, Tang S, Jiang N, Cui L, Zhao N, Lin Q, Chen L, Lin H, Jin X, Dong Z, Ren Z, Hou Z, Zhang Y, Zhong J, Cai S, Liu Y, Meng R, Deng Y, Ding X, Ma J, Xie Z, Shen L, Wu W, Zhang M, Ying Q, Zeng Y, Dong J, Cummings SR, Li Z, Xia W (2021). Prevalence of osteoporosis and fracture in China: the China osteoporosis prevalence study. JAMA Netw Open.

[4] Gruber R, Koch H, Doll BA, Tegtmeier F, Einhorn TA, Hollinger JO (2006). Fracture healing in the elderly patient. Exp Gerontol.

[5] Duque G, Huang DC, Dion N, Macoritto M, Rivas D, Li W, Yang XF, Li J, Lian J, Marino FT, Barralet J, Lascau V, Deschênes C, Ste-Marie LG, Kremer R (2011). Interferon-γ plays a role in bone formation in vivo and rescues osteoporosis in ovariectomized mice. J Bone Miner Res.

[6] Walsh WR, Sherman P, Howlett CR, Sonnabend DH, Ehrlich MG (1997). Fracture healing in a rat osteopenia model. Clin Orthop Relat Res.

[7] Shi C, Wu J, Yan Q, Wang R, Miao D (2015). Bone marrow ablation demonstrates that estrogen plays an important role in osteogenesis and bone turnover via an antioxidative mechanism. Bone.

[8] Meyer MH, Meyer RA (2006). Altered expression of mitochondrial genes in response to fracture in old rats. Acta Orthop.

[9] Tatehara S, Miyamoto Y, Takechi M, Momota Y, Yuasa T (2011). Osteoporosis influences the early period of the healing after distraction osteogenesis in a rat osteoporotic model. J Craniomaxillofac Surg.

[10] Deng C, Wynshaw-Boris A, Zhou F, Kuo A, Leder P (1996). Fibroblast growth factor receptor 3 is a negative regulator of bone growth. Cell.

[11] Rousseau F, Bonaventure J, Legeai-Mallet L, Pelet A, Rozet JM, Maroteaux P, Le Merrer M, Munnich A (1994). Mutations in the gene encoding fibroblast growth factor receptor-3 in achondroplasia. Nature.

[12] Kim SJ, Pierce W, Sabharwal S (2014). The etiology of short stature affects the clinical outcome of lower limb lengthening using external fixation. A systematic review of 18 trials involving 547 patients. Acta Orthop.

[13] Osawa Y, Matsushita M, Hasegawa S, Esaki R, Fujio M, Ohkawara B, Ishiguro N, Ohno K, Kitoh H (2017). Activated FGFR3 promotes bone formation via accelerating endochondral ossification in mouse model of distraction osteogenesis. Bone.

[14] Su N, Sun Q, Li C, Lu X, Qi H, Chen S, Yang J, Du X, Zhao L, He Q, Jin M, Shen Y, Chen D, Chen L (2010). Gain-of-function mutation in FGFR3 in mice leads to decreased bone mass by affecting both osteoblastogenesis and osteoclastogenesis. Hum Mol Genet.

[15] Naski MC, Colvin JS, Coffin JD, Ornitz DM (1998). Repression of hedgehog signaling and BMP4 expression in growth plate cartilage by fibroblast growth factor receptor 3. Development.

[16] Fujio M, Yamamoto A, Ando Y, Shohara R, Kinoshita K, Kaneko T, Hibi H, Ueda M (2011). Stromal cell-derived factor-1 enhances distraction osteogenesis-mediated skeletal tissue regeneration through the recruitment of endothelial precursors. Bone.

[17] Gdalevitch M, Kasaai B, Alam N, Dohin B, Lauzier D, Hamdy RC (2013). The effect of heparan sulfate application on bone formation during distraction osteogenesis. PLoS ONE.

[18] Troulis MJ, Coppe C, O’Neill MJ, Kaban LB (2003). Ultrasound: assessment of the distraction osteogenesis wound in patients undergoing mandibular lengthening. J Oral Maxillofac Surg.

[19] Mishima K, Kitoh H, Ohkawara B, Okuno T, Ito M, Masuda A, Ishiguro N, Ohno K (2015). Lansoprazole upregulates polyubiquitination of the TNF receptor-associated factor 6 and facilitates Runx2-mediated osteoblastogenesis. EBioMedicine.

[20] Perrien DS, Nicks KM, Liu L, Akel NS, Bacon AW, Skinner RA, Swain FL, Aronson J, Suva LJ, Gaddy D (2012). Inhibin a enhances bone formation during distraction osteogenesis. J Orthop Res.

[21] Stine KC, Wahl EC, Liu L, Skinner RA, Vanderschilden J, Bunn RC, Montgomery CO, Suva LJ, Aronson J, Becton DL, Nicholas RW, Swearingen CJ, Lumpkin CK (2014). Cisplatin inhibits bone healing during distraction osteogenesis. J Orthop Res.

[22] Ikuta K, Urakawa H, Kozawa E, Hamada S, Ota T, Kato R, Honda H, Kobayashi T, Ishiguro N, Nishida Y (2015). In vivo heat-stimulus-triggered osteogenesis. Int J Hyperthermia.

[23] Akagi H, Ochi H, Soeta S, Kanno N, Yoshihara M, Okazaki K, Yogo T, Harada Y, Amasaki H, Hara Y. A comparison of the process of remodeling of hydroxyapatite/Poly-D/L-lactide and beta-tricalcium phosphate in a loading site. BioMed Res Int. 2015;730105. 10.1155/2015/730105. 10.1155/2015/730105 PMC4609391 26504825

[24] Dempster DW, Compston JE, Drezner MK, Glorieux FH, Kanis JA, Malluche H, Meunier PJ, Ott SM, Recker RR, Parfitt AM (2013). Standardized nomenclature, symbols, and units for bone histomorphometry: a 2012 update of the report of the ASBMR histomorphometry nomenclature Committee. J Bone Miner Res.

[25] Matsushita M, Kitoh H, Ohkawara B, Mishima K, Kaneko H, Ito M, Masuda A, Ishiguro N, Ohno K (2013). Meclozine facilitates proliferation and differentiation of chondrocytes by attenuating abnormally activated FGFR3 signaling in achondroplasia. PLoS ONE.

[26] Matsushita M, Esaki R, Mishima K, Ishiguro N, Ohno K, Kitoh H (2017). Clinical dosage of meclozine promotes longitudinal bone growth, bone volume, and trabecular bone quality in transgenic mice with achondroplasia. Sci Rep.

[27] Luo Z, Liu M, Sun L, Rui F (2015). Icariin recovers the osteogenic differentiation and bone formation of bone marrow stromal cells from a rat model of estrogen deficiency-induced osteoporosis. Mol Med Rep.

[28] Biosse Duplan M, Dambroise E, Estibals V, Veziers J, Guicheux J, Legeai-Mallet L (2021). An FGFR3-activating mutation in immature murine osteoblasts affects the appendicular and craniofacial skeleton. Dis Model Mech.

[29] Abzhanov A, Rodda SJ, McMahon AP, Tabin CJ (2007). Regulation of skeletogenic differentiation in cranial dermal bone. Development.

[30] Szabova L, Yamada SS, Wimer H, Chrysovergis K, Ingvarsen S, Behrendt N, Engelholm LH, Holmbeck K (2009). MT1-MMP and type II collagen specify skeletal stem cells and their bone and cartilage progeny. J Bone Miner Res.

[31] Su N, Li X, Tang Y, Yang J, Wen X, Guo J, Tang J, Du X, Chen L (2016). Deletion of FGFR3 in osteoclast lineage cells results in increased bone mass in mice by inhibiting osteoclastic bone resorption. J Bone Miner Res.

[32] Wang LC, Takahashi I, Sasano Y, Sugawara J, Mitani H (2005). Osteoclastogenic activity during mandibular distraction osteogenesis. J Dent Res.

[33] Guo J, Li W, Wu Y, Jing X, Huang J, Zhang J, Xiang W, Ren R, Lv Z, Xiao J, Guo F (2017). Meclizine prevents ovariectomy-induced bone loss and inhibits osteoclastogenesis partially by upregulating PXR. Front Pharmacol.

