# Supplementary material for: Activated FGFR3 suppresses bone regeneration and bone mineralization in an ovariectomized mouse model
Source: BMC Musculoskelet Disord. 2023 Mar 16;24:200. doi: 10.1186/s12891-023-06318-9 (PMC10018961; doi:10.1186/s12891-023-06318-9)
Supplement: Supplementary file 1 — Supplementary Material 1 [file 12891_2023_6318_MOESM1_ESM.pdf]

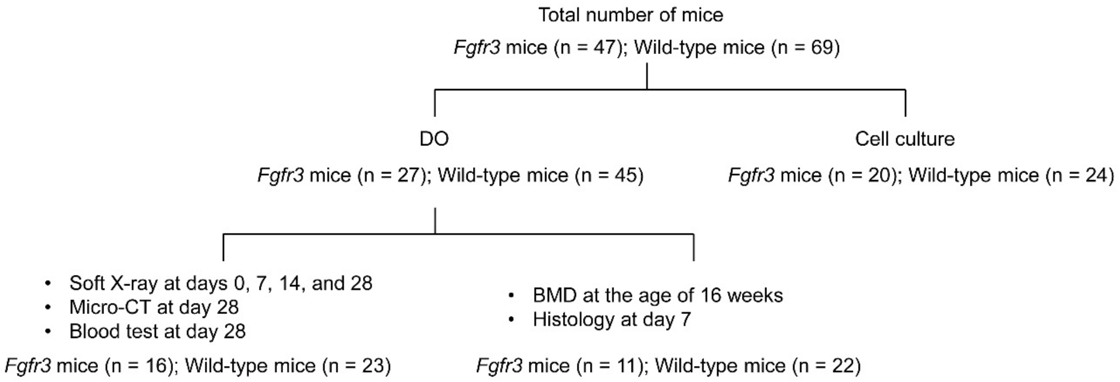

Additional Figure 1 Number of *Fgfr3* mice and wild-type mice. The mice were subjected to distraction osteogenesis (DO) surgery or cell culture. We performed soft X-ray, micro-CT scan, and blood test in the same individual for 16 *Fgfr3* mice and 23 wild-type mice. Bone mineral density (BMD) and histology were evaluated in the same individual for 11 *Fgfr3* mice and 22 wild-type mice.

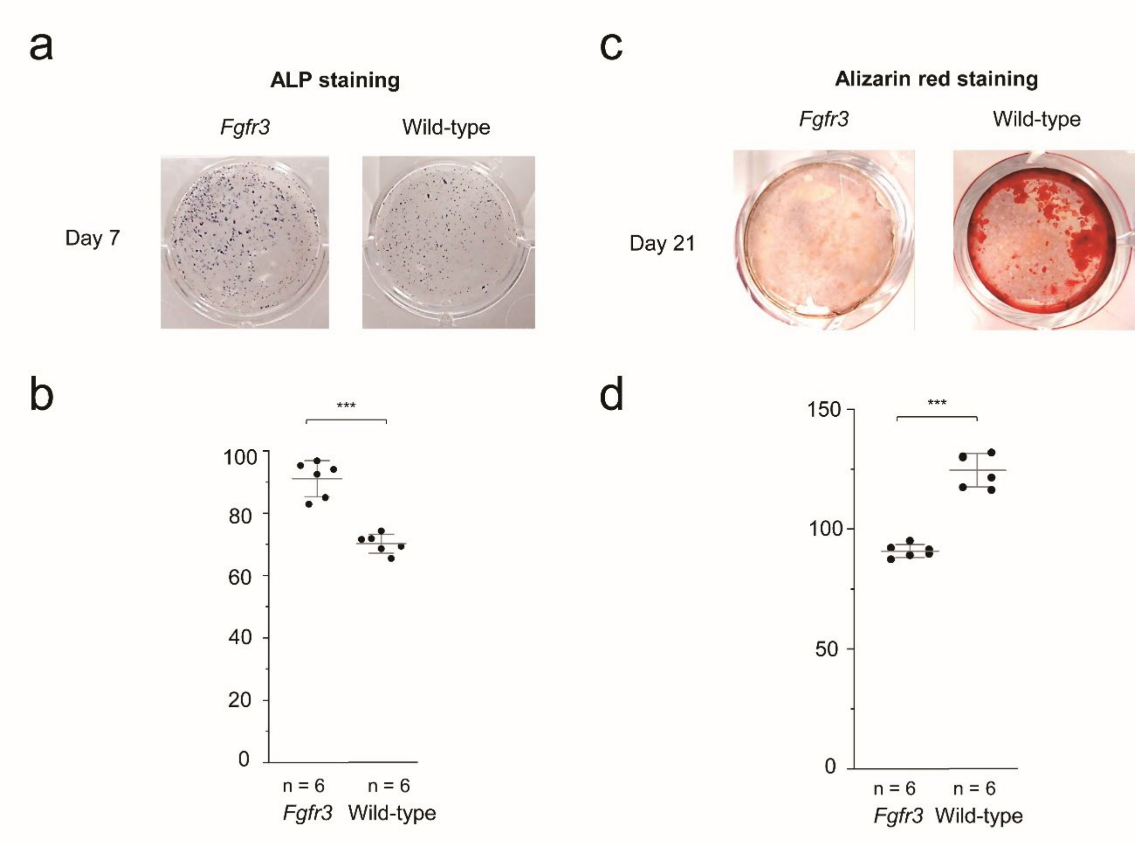

Additional Figure 2 Osteogenic differentiation and mineralization of bone marrow-derived mesenchymal stem cells in 4-week-old mice. **a** Representative result of alkaline phosphatase (ALP) staining, at the seventh day of the culture in 4-week-old mice. **b** Quantitative analysis of ALP staining. **c** Representative result of Alizarin red staining of the mineralized osteoblasts. **d** Quantitative analysis of Alizarin red staining. Dots indicate the value of each sample and bars indicate the means and standard deviations. Statistical significance was analyzed using Student's t-test. Statistical significance was expressed as *** $p < 0.005$.

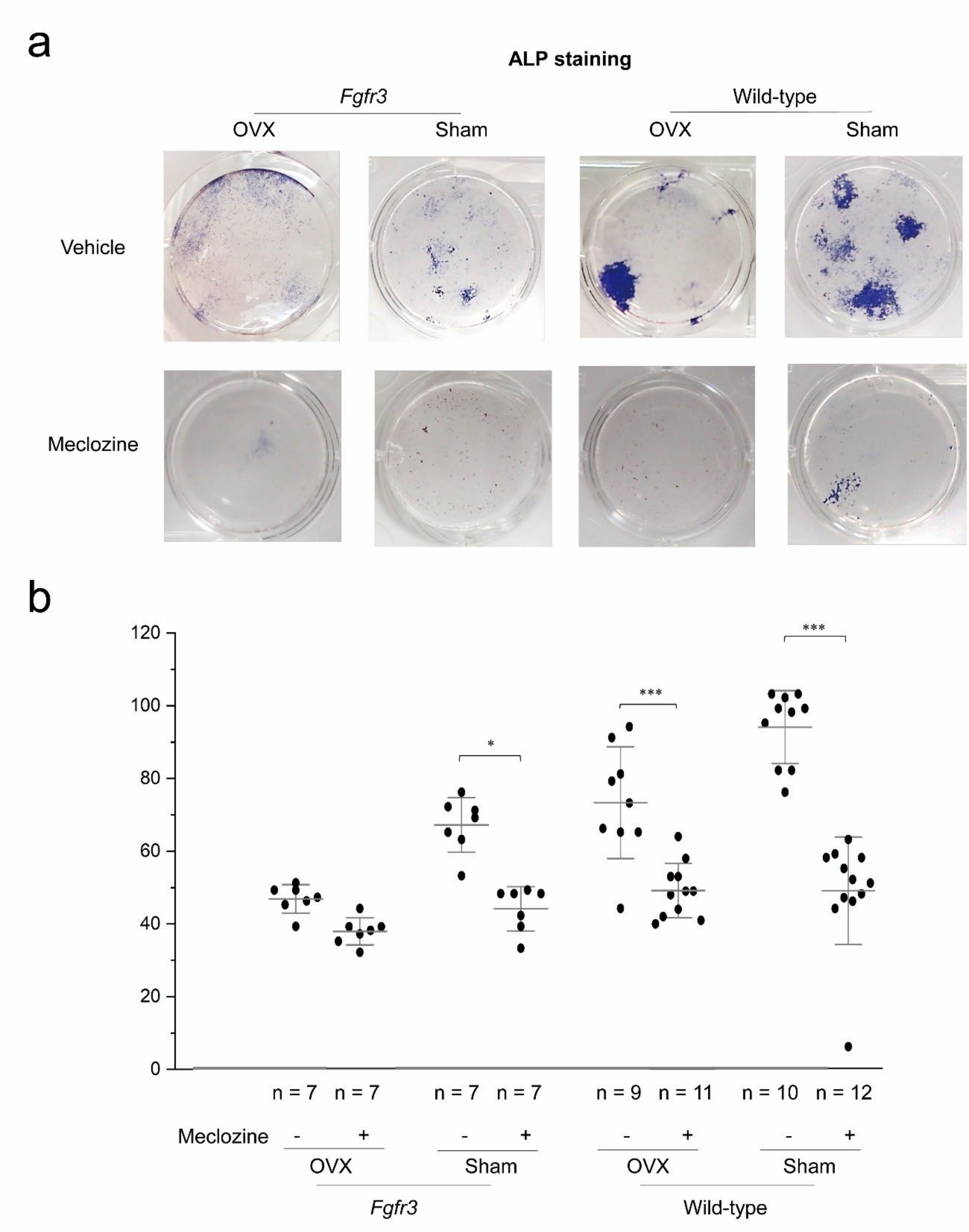

Additional Figure 3 Osteogenic differentiation of bone marrow-derived mesenchymal stem cells (BMSCs) in each group. **a** Representative result of alkaline phosphatase (ALP) staining for cultured BMSCs with meclozine treatment. **b** Quantitative analysis of ALP staining to assess the differentiation of BMSCs. Dots indicate the value of each sample and bars indicate the means and SD. Statistical significance was analyzed using one-way analysis of variance (ANOVA) with post-hoc Bonferroni correction. Statistical significance was expressed as * $p < 0.05$ and *** $p < 0.005$.
